# Spinal Anesthesia for Ambulatory Surgery Using Hyperbaric Prilocaine vs Hyperbaric Bupivacaine: A Prospective Study

**DOI:** 10.7759/cureus.78246

**Published:** 2025-01-30

**Authors:** Nelson Gomes, Ana Castro, Sandra Borges, Paula Sarmento

**Affiliations:** 1 Anesthesiology, Unidade Local de Saúde de Entre Douro e Vouga, Santa Maria da Feira, PRT

**Keywords:** ambulatory surgery, bupivacaine, operation room efficiency, perioperative complication, prilocaine, spinal anesthesia

## Abstract

Background

Spinal anesthesia is a suitable technique for ambulatory surgery, offering benefits such as rapid recovery and minimal complications. The choice of local anesthetic is a key factor in achieving a rapid turnover in an ambulatory surgery unit. This study compares the safety and effectiveness of hyperbaric prilocaine and hyperbaric bupivacaine in an ambulatory setting.

Methods

A total of 114 patients undergoing ambulatory surgery were randomly allocated to receive either hyperbaric prilocaine (20mg/mL) or hyperbaric bupivacaine (5mg/mL). Time to first walk, time to discharge, and perioperative complications were compared.

Results

Eighty-nine patients were analyzed: the median time to first unassisted ambulation was significantly reduced by 41 minutes in the prilocaine group, and the average discharge time was reduced by 29 minutes. Hypotension was the most frequently observed complication, occurring in 17.4% of the prilocaine group and 16.3% of the bupivacaine group, with no statistically significant difference. There was no statistically significant difference between the two groups in other perioperative complications studied.

Conclusion

Hyperbaric prilocaine is a safe and effective alternative to hyperbaric bupivacaine for ambulatory surgery, offering a rapid onset and intermediate duration, resulting in earlier patient discharge. Improving the perioperative times can make the ambulatory unit more efficient and contribute to cost reduction.

## Introduction

Ambulatory surgery continues to grow within healthcare services [1], and the quest for an ideal anesthetic technique for outpatient surgery persists. The optimal strategy should be easy to apply, have a predictable onset and duration, and minimize adverse effects [2,3]. Spinal anesthesia (SPA) is a safe and versatile technique that suits the requirements of ambulatory surgery [1,2]. It offers multiple advantages, such as the avoidance of mechanical ventilation and airway challenges, a reduced risk of residual effects from hypnotics and opioids, decreased blood loss, a lower incidence of postoperative nausea and vomiting (PONV), improved pain control, and a faster recovery [2-4]. However, potential risks associated with the use of SPA include post-dural puncture headache (PDPH), transient neurologic symptoms (TNS), and postoperative urinary retention (POUR) [2-5].

The selection of a local anesthetic for SPA primarily depends on the anticipated duration of the surgical procedure [1-2,5]. Prilocaine appears to be ideal for outpatient SPA due to its pharmacological properties (rapid onset, intermediate duration, and potency), good safety profile (low incidence of TNS), and economic acceptability [1-4]. In one-day cases, bupivacaine, which has a longer duration of action, is more difficult to manage and can lead to prolonged discharge times [2-5]. Delivering high-quality healthcare services in ambulatory surgery requires efficient processes to ensure long-term financial viability [4]. Recently, we have implemented several changes in our ambulatory unit, including introducing prilocaine among our local anesthetic options.

This study aims to compare the time taken to first walk and the time taken to discharge when hyperbaric prilocaine and hyperbaric bupivacaine are utilized in one-day surgery. Secondary aims include comparing complications and the requirement for additional analgesia.

## Materials and methods

This randomized trial was performed between September and December 2022 at the Ambulatory Surgery Center of Unidade Local de Saúde de Entre Douro e Vouga, Portugal.

The sample size for this study was calculated using the formula: n = 2 [(Zα + Z1-β)²] * σ² / δ², where n represents the sample size for each group, Zα is a constant set by convention according to the accepted alpha error, Z(1-β) is a constant set by convention according to the study's power, σ is the standard deviation, and δ is the effect size. A standard deviation of 42 minutes was considered based on the findings of Wesselink et al. [6], assuming a mean difference of 30 minutes, with a power of 80% and a significance level of 0.05. Taking into account a 20% attrition rate due to patient dropout, the calculated sample size was 78 participants (39 per group). After reaching our target sample size, we decided to continue the study until the end of the period we had initially defined.

The inclusion criteria were ages between 18 and 85, American Society of Anesthesiologists (ASA) physical status class I, II, or III, eligibility for outpatient surgery, and meeting criteria for SPA.

Exclusion criteria were patients with contraindications to SPA, allergy or hypersensitivity to the studied local anesthetics, use of intrathecal opioids, conversion to general anesthesia, and planned overnight stay.

The following parameters were recorded: gender, age, body mass index (BMI), ASA physical status, local anesthetic doses, surgical specialty, surgical procedure, surgery duration, time of first walk without assistance, time to discharge, perioperative complications (hypotension, bradycardia, nausea and vomiting, urinary retention, post-dural puncture headache, transient neurologic symptoms), rescue analgesia and pain assessment at 24 hours.

Study design

A sealed envelope system was used to randomly allocate patients into two different groups with a 1:1 ratio: Group P (experimental group, Takipril® hyperbaric prilocaine, BBraun, Germany, 20mg/ml) and Group B (control group, Bupinostrum® hyperbaric bupivacaine, Bluemed, Portugal, 5mg/ml). The envelope contained the name of the local anesthetic to be used in spinal anesthesia, as well as the recommended dose for the surgical procedure according to the recommendations for regional anesthesia in outpatient surgery of the Associação Portuguesa de Cirurgia Ambulatória (APCA, Portuguese Ambulatory Surgery Association).

The anesthesiologist in charge of the case opened the envelope prior to the patient's entry into the operating room. Both the anesthesiologist and the anesthesia nurse were not blinded and were responsible for completing the anesthesia record during the intraoperative period. This record was accessible only to them, and they maintained the documentation throughout the intraoperative and postoperative phases until the patient was discharged. The record was added to the patient's medical file at that point. The remaining nursing team (both intraoperative and post-anesthesia care unit (PACU)), the surgeons, the patient, and the person responsible for the statistics were blinded. The SPA was performed by the anesthesiologist handling the case, while clinical assessments and recovery times after surgery were conducted by the PACU nurses. If any serious anesthetic or surgical complications occurred, the blinding would be broken, and the patient would be excluded from the study.

At admission to the operating theatre, an intravenous cannula was inserted, and patients were premedicated with 1mg midazolam IV, 10mg metoclopramide IV, and 40mg pantoprazole IV. A 500ml isotonic crystalloid solution (Plasma-lyte®) was used for intravenous perfusion, being discontinued during PACU stay.

In the operating room, patients were monitored according to ASA monitoring standards, and measurements were recorded every 5 minutes. Spinal anesthesia was performed at the L3-L4 intervertebral spaces, with patients placed in the lateral decubitus position. A midline or lateral approach was used with a 27G Quincke spinal needle. After verifying the free flow of clear cerebrospinal fluid, the local anesthetic was injected into the intrathecal space over 10-20 seconds. Patients were placed supine after the injection.

To ensure the level of sensory blockade was adequate for a painless surgery, we used a standard cold spray sensation test. Before making the skin incision, the surgeon also performed a pinprick test at least 2 to 3 segments higher than necessary. At the end of the surgery, patients were transferred to the PACU. As the motor block started diminishing, patients received 1g paracetamol IV and 30mg ketorolac IV (or 20mg for patients 65 years and older) as part of our standard analgesia protocol.

Patients were required to have a score of at least 9 out of 10 on the Modified Postanesthetic Discharge Score System (MPADSS) to ensure recovery and leave the PACU for phase II recovery room. At that stage, the visual analogue scale (VAS) was used to assess pain; if VAS > 3/10, 2g of metamizole IV was administered as rescue analgesia. Persistent pain would be treated with 50-100mg of tramadol IV.

To be safely discharged home, patients must demonstrate stability in terms of vital signs, pain, nausea, and vomiting. They must also exhibit the ability to stand and walk with minimal or similar assistance before the procedure. We utilized the Bromage scale to verify complete recovery from the motor block. Being able to urinate was necessary to be discharged. If the patient was unable to void, the patient would be evaluated for urinary retention and the need for urinary catheterization.

Patients were discharged from the unit medicated with paracetamol 1g and ibuprofen 400mg orally every 8-8 hours. The day after surgery, all patients were contacted by telephone and asked about pain and analgesic use, low back pain, headache and transient neurological symptoms. One to two weeks after surgery, patients were evaluated by the surgical team and the existence of any complications was recorded.

Perioperative complications

Perioperative complications were evaluated according to the following definitions: hypotension (≥ 20% decrease in mean arterial blood pressure compared to the baseline, or systolic arterial pressure < 90mmHg or use vasopressor drugs), bradycardia (< 40 beats/min or use atropine IV), nausea and vomiting (or use of antiemetics), POUR (inability to void within 4h after surgery and need urinary catheterization to empty the bladder), PDPH (frontal or occipital headache, or neck pain, worse when standing and better when lying down, starting one day to one week after spinal technique), TNS (pain, dysthyesia, or both in buttocks and/or lower extremities; appears within 24 hours of spinal anesthesia, lasts 2-5 days, and resolves completely without sequelae), pain assessment at 24 hours (controlled pain if VAS <4).

Statistical analysis

Categorical variables are presented as frequencies and percentages, and continuous variables are presented as means and standard deviations or medians and interquartile ranges for variables with skewed distributions. Normal distribution was checked using the Shapiro-Wilk test or skewness and kurtosis. Differences and associations of qualitative variables were tested with the Chi-square test or Fisher's exact test, and differences between quantitative variables using the t-student test or Mann-Whitney U test, as appropriate. All reported P values are two-tailed, with a P value of 0.05 indicating statistical significance. Data was analyzed by an independent person without medical knowledge, using the IBM SPSS Statistics for Windows, version 26.0.

Ethics statement

This study received approval from the Hospital Ethical Committee (approval number: CA-0057/2022-0t_MP/CC), and all selected patients signed a written informed consent to participate.

## Results

**Figure 1:**
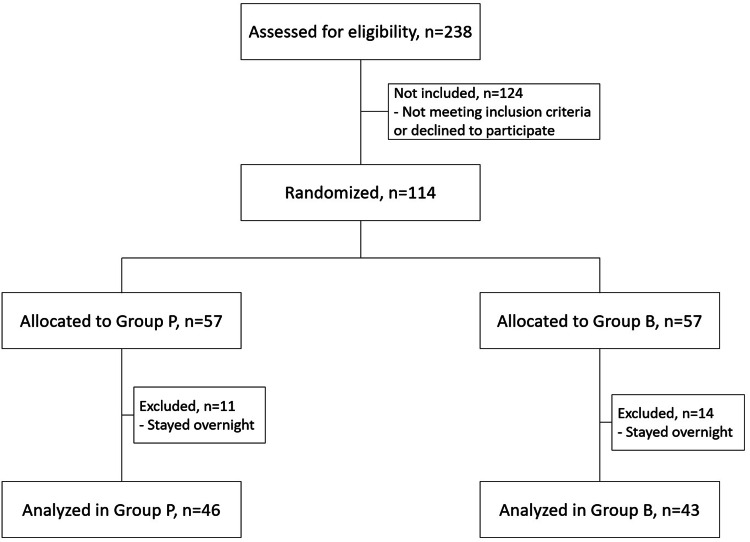
CONSORT flow chart diagram of the study design.

Of the initial 114 patients, 25 were excluded from the final analysis due to overnight stays; there were no cases of failed spinal block (Figure 1).

Eighty-nine patients were analyzed: 46 in Group P and 43 in Group B. Patients' characteristics were similar between the groups and are summarized in Table 1.

**Table 1:** Demographic characteristics of patients. n - number of participants; * Mean (± standard deviation); ASA - American Society of Anesthesiologists.

Variables		Prilocaine (n=46)	Bupivacaine (n=43)	p-value
Gender	Female (n, %)	11 (24%)	7 (16%)	0.370
	Male (n, %)	35 (76%)	36 (84%)	
Age* (yrs)		52.4 (±12.4)	57.6 (±14.4)	0.072
Body Mass Index* (kg/m2) Prilocaine (n=44), bupivacaine (n=40)		27.0 (±3.5)	26.7 (±3.4)	0.741
ASA physical status	I	3 (6.5%)	4 (9.3%)	0.296
	II	42 (91.3%)	35 (81.4%)	
	III	1 (2.2%)	4 (9.3%)	

The most common surgical specialty was general surgery with 78.7% of all surgeries; surgical specialties had a similar proportion of cases between groups (p=0.544). Surgical specialties and procedures are outlined (Tables 2-3), respectively.

**Table 2:** Surgical specialties. n - number of participants.

Surgical specialties	Prilocaine (n=46)	Bupivacaine (n=43)	p-value
General Surgey	37 (80.4%)	33 (76.7%)	0.544
Orthopedics	8 (17.4%)	6 (14%)	
Urology	1 (2.2%)	2 (4.7%)	
Gynecology	0 (0%)	2 (4.7%)	

**Table 3:** Surgical procedures. n - number of participants.

Procedures	Prilocaine (n=46)	Bupivacaine (n=43)	p-value
Anal fistulotomy	2 (4.3%)	1 (2.3%)	0.172
Hallux valgus surgery	0 (0%)	2 (4.7%)	
Hemorrhoidectomy	9 (19.6%)	4 (9.3%)	
Hydrocelectomy	0 (0%)	1 (2.3%)	
Inguinal hernia repair	19 (41.3%)	24 (55.8%)	
Knee arthroscopy	7 (15.2%)	4 (9.3%)	
Knee osteosynthesis material extraction	1 (2.2%)	0 (0%)	
Penis repair	1 (2.2%)	0 (0%)	
Pilonidal cyst removal	5 (10.9%)	1 (2.3%)	
Rectal polyp excision	0 (0%)	1 (2.3%)	
Tension-free vaginal tape surgery	0 (0%)	2 (4.7%)	
Umbilical hernia repair	1 (2.2%)	2 (4.7%)	
Vasectomy	1 (2.2%)	0 (0%)	
Varicose vein stripping and ligation	1 (2.2%)	0 (0%)	

The median surgical duration was similar between groups (50 minutes, p=0.934, Table 4). The median dose used for surgical procedures was 45mg in group P (interquartile range: 40-50mg; minimum 25mg-maximum 55mg) and 9mg in group B (interquartile range: 8-9mg; minimum 5mg-maximum 10mg).

**Table 4:** Comparison of groups in terms of perioperative events and complications, rescue analgesia, surgical time and postoperative recovery times. n – number of participants; PONV – postoperative nausea and vomiting; POUR – postoperative urinary retention; PDPH – post-dural puncture headache; # Median [interquartile range, (minimum-maximum)]; * Mean (± standard deviation). The time until the first walk and the time until discharge were measured from the moment the spinal anesthesia technique was performed.

Variable	Prilocaine (n=46)	Bupivacaine (n=43)	p-value
Hypotension	8 (17.4%)	7 (16.3%)	0.889
Bradycardia	4 (8.7%)	3 (7%)	1.000
PONV	4 (8.7%)	6 (14%)	0.513
POUR	1 (2.2%)	0 (0%)	1.000
Metamizole	12 (26.1%)	4 (9.3%)	0.039
Tramadol	1 (2.2%)	0 (0%)	1.000
PDPH	1 (2.2%)	0 (0%)	1.000
Surgical time# (min)	50 [42.25-60, (25-105)]	50 [40-69, (30-100)]	0.934
Postanesthesia phase I time# (min)	89.5 (63-116.5, (35-175)]	90 [67-115, (28-180)]	0.669
Time to first walk# (min)	246.50 [203.75-300, (135-447)]	287 [247-340, (87-446)]	0.008
Time to discharge* (min)	336.13 (±69.25)	365.44 (±59.76)	0.036

Regarding perioperative events, the most common complication was hypotension (17.4% in group P versus 16.3% in group B), with no significant differences between groups (p=0.899). The remaining events (bradycardia, PONV, POUR) also exhibited no significant differences between groups (Table 4).

There was a greater need for rescue analgesia in the Group P compared to the Group B (26.1% vs 9.3, p=0.039, Table 4). The median length of stay in phase I recovery was similar between groups (89.5 minutes in Group P vs. 90 minutes in Group B, p=0.669).

In the post-anesthetic phase II area, there was a difference in the median time to first unassisted ambulation, approximately 40.5 minutes earlier in the Group P (246.5 vs 286 minutes, p=0.008). The average time to discharge was 29 minutes shorter in Group P (336.13 vs 365.44 minutes, p=0.036). The postoperative recovery times are summarized in Table 4.

There was no significant difference in PDPH between groups (Table 4). No cases of low back pain and TNS were reported. The patients who responded to our 24-hour assessment call had controlled pain [(Group P, 35 patients (76.1%); Group B, 39 patients (90.7%)].

## Discussion

Our study demonstrated that prilocaine significantly reduced both the time to first walk and the time to discharge, thereby decreasing the overall duration of patients' stay in the outpatient facility. Compared to our standard practice, prilocaine was safe and widely used drug, with no incidents of unplanned admissions related to anesthetic complications. Overnight stays were primarily attributed to late-scheduled surgeries, considerable distances from the hospital, or lack of a caregiver during the immediate post-discharge period.

The median time to unassisted ambulation in group P was 246.5 minutes, aligning with findings from similar studies using comparable doses [7,8]. However, studies utilizing 50mg and 45mg of prilocaine reported shorter times of 204.5 minutes [9] and 178.5 minutes [10], respectively. We reported a mean discharge time of 336.13 minutes, consistent with most studies [7,8,11]. Although, some studies have reported shorter times until hospital discharge: 198.5 [10], 256 [12], and 282 [6] minutes. We hypothesize that mandatory bladder voiding may have increased our time to discharge [13,14].

Hypotension is a relatively common adverse effect with SPA [3,15]. We found no significant differences between groups, with hypotension occurring in 17.4% of group P, within the 5-22% range reported in the literature [8,11,16]. Bradycardia was observed in 8.7% of patients in group P, slightly higher than the 4.8-7.5% reported in some recent studies [11,16]. Despite periods of hypotension and bradycardia, our patients did not experience any morbidity. The incidence of PONV was 8.7%, which is above the 1.1-7.8% range reported in previous studies [6,10,11]. This difference may be attributed to the routine administration of antiemetics during SPA by some anesthesiologists involved in the study, in addition to metoclopramide, which was part of the protocol.

Inability to void can lead to bladder distension, urinary tract infection, and potentially kidney dysfunction [3,4]. We observed one case of POUR, corresponding to 2.2%, similar to Ambrosoli's retrospective study reporting 1.09% in 3291 patients [1] and within the literature range of 0 to 23.3% [6,10,11,13,17]. This case involved a male with benign prostatic hyperplasia who underwent hemorrhoidectomy surgery, both recognized risk factors for POUR [13,14]. Additionally, we report one case of PDPH (2.2%) in a 38-year-old woman treated conservatively. Studies report PDPH values of 0-3.1% [1,6,8,11]. We observed no cases of TNS or low back pain; TNS is rarely associated with prilocaine, with most studies showing no cases [11,16] or negligible occurrence (~0.03%) [1]. Complaints of pain at the puncture site or lower back pain are also infrequent, ranging from 0 to 10% [11,12].

We found a difference in analgesia requirements during the recovery phase, likely due to prilocaine's shorter sensory blockade duration, which may have prompted earlier analgesic use, as noted in previous studies [18,19].

Limitations

We acknowledge some limitations in our study. First, a considerable proportion of patients stayed overnight. Second, there was variability in surgical specialties and the doses administered for different procedures. Third, a recent modification in the internal protocol for patients undergoing spinal anesthesia may have affected discharge times, as some nursing team members were unfamiliar with the discharge scoring systems.

## Conclusions

Spinal anesthesia remains a valid approach in the ambulatory setting. Hyperbaric prilocaine is a safe drug with a rapid onset and intermediate duration, resulting in earlier patient discharge. Improving the perioperative times due to faster turnovers can significantly impact the organization of the outpatient unit and contribute to cost reduction.
